# Adenovirus Vector‐Mediated In Vivo Knock‐in Treatment of Neonatal Phenylketonuria Mice Using Terminally Cleaved Donor DNA

**DOI:** 10.1002/jgm.70104

**Published:** 2026-08-11

**Authors:** Megumi Yamaji, Hirotaka Tabata, Mariko Nakamura, Ryoko Fuse, Tadashi Okada, Shun‐Ichi Ohba, Tomokazu Ohishi, Manabu Kawada, Izumu Saito, Shigeto Sato, Tomoko Nakanishi

**Affiliations:** ^1^ Center for Biomedical Research Resources Juntendo University Graduate School of Medicine Tokyo Japan; ^2^ Department of Pharmaceutical Engineering, Faculty of Engineering Toyama Prefectural University Toyama Japan; ^3^ Laboratory of Virology Institute of Microbial Chemistry (BIKAKEN), Microbial Chemistry Foundation Tokyo Japan; ^4^ Department of Food Management Okayama Gakuin University Okayama Japan; ^5^ Institute of Microbial Chemistry (BIKAKEN), Numazu, Microbial Chemistry Research Foundation Shizuoka Japan; ^6^ Department of Physiology Juntendo University Graduate School of Medicine Tokyo Japan

**Keywords:** adenovirus vector (AdV), Cas9 nickase, knock‐in, multiplex gRNA, phenylalanine hydroxylase (PAH), phenylketonuria (PKU)

## Abstract

**Background:**

Adenovirus vectors (AdVs) are widely used and have an advantage of large insert capacity compared with adeno‐associated virus vectors. However, AdVs have scarcely been used in genome‐editing knock‐in strategies because of low efficiency.

**Methods:**

Novel AdVs possessing a very large, 3.7 kb donor DNA fragment and six or eight multiplex gRNA expression units were developed for CRISPR/Cas9‐mediated knock‐in to correct a phenylalanine hydroxylase (*Pah*) gene in a *Pah* ^enu2^ phenylketonuria mouse model. These AdVs were co‐infected to Hepa1–6 cells or liver cells in vivo together with an AdV expressing either native Cas9 or Cas9 nickase (Cas9n) for double‐nicking cleavage.

**Results:**

In vitro knock‐in of the AdVs carrying 3.7 kb donor DNA and six gRNA units targeting the cell genome was observed in both cases using Cas9 and Cas9n, though their efficiencies were low. Therefore, we generated AdVs carrying an additional two gRNA units that cleave the donor DNA terminus in the AdV genome via native Cas9 or Cas9 nickase. The knock‐in efficiency increased approximately twofold for both vectors and reached a maximum of 8% for native Cas9 without selection. Newborn phenylketonuria model mice were intravenously administered the knock‐in AdV together with the native‐Cas9 AdV. Although the knock‐in efficiency by homologous recombination occurred in only approximately 1% of hepatocytes, blood phenylalanine levels were reduced by up to 30%. Also, unintended fragments produced by nonhomologous end‐joining were observed between the cleavage site at the terminus of the donor DNA in the AdV genome and the target site in the cell genome.

**Conclusions:**

The knock‐in efficiency of AdVs can be increased by cleaving the terminus of the donor DNA, although it would be desirable to avoid nonhomologous end‐joining between double‐strand break termini.

## Graphical abstract

We successfully developed a highly efficient knock‐in adenovirus vector, AxPah‐6g‐cut. This vector cleaves not only the genomic target site but also the terminus of the donor DNA in the vector. When this vector was intravenously administered twice to phenylketonuria (*Pah* ^enu2^) mice together with a Cas9‐expressing vector, AxCBCas9, it resulted in the modest improvement of hyperphenylalaninemia.

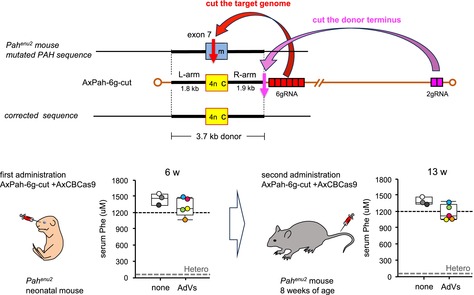

**Abbreviations**
AdV: adenovirus vector
Cas9n: Cas9 nickase
gRNA: guide RNA
MOI: multiplicity of infection
PAH (Pah): phenylalanine hydroxylase
PKU: phenylketonuria
TIDER: tracking of insertions deletions and end‐joining repair

## 1 Introduction

Phenylketonuria (PKU) is an inherited, autosomal recessive metabolic disorder caused by variations in the phenylalanine hydroxylase (*PAH*) gene that leads to PAH deficiency. PAH is essential for the metabolism of phenylalanine to tyrosine. A lack of functional PAH leads to chronic hyperphenylalaninemia, which can cause severe neurological damage, leading to intellectual disability, psychological disorders, and seizures [1, 2, 3, 4, 5, 6]. The *PAH* gene is located on chromosome 12q22‐q24.1, contains 13 exons and 12 introns, and encodes a 452–amino acid protein. A broad spectrum of *PAH* gene mutations is responsible for PKU, with over 500 distinct *PAH* variations identified to date (http://www.biopku.org/pah/home.asp) [7, 8].

CRISPR/Cas9 technology is one of the most promising tools for gene modification and the treatment of genetic diseases. The nuclease Cas9 efficiently induces double‐strand breaks at DNA target sites under the direction of gRNA [9, 10]. Site‐specific double‐strand breaks induce two major repair mechanisms: nonhomologous end‐joining (NHEJ) and homology‐directed repair (HDR) [11, 12]. HDR‐mediated gene repair can correct loss‐of‐function mutations in adult animal disease models, and a PKU mouse model has been treated by exon replacement via homologous recombination‐mediated knock‐in [13]. Moreover, a variety of CRISPR/Cas9 system‐based gene‐editing strategies, including prime editing and base editing, have been used to treat certain point mutations [14, 15, 16].

Viral vectors are attractive gene delivery vehicles because they can efficiently transfer a gene to a wide variety of cell types [17, 18]. Adeno‐associated virus (AAV) vectors are currently the most widely used, but their packaging capacity is limited to approximately 4.5 kb. This limitation restricts the length of homology arms, which are critical for achieving high‐efficiency gene knock‐in. Additionally, AAV vectors are associated with a high frequency of random genomic integration, which is a safety concern. Furthermore, their production costs are high [19, 20]. In contrast, adenovirus vectors (AdV) possess a large packaging capacity of up to 8 kb. AdVs are also beneficial as a result of their minimal integration into the host genome [19, 21] and their capacity for easy, large‐scale, and cost‐effective production [22]. Although the high immunogenicity of AdV has been a major concern for its use in gene therapy [23, 24], it has been reported that AdV‐induced immune responses are primarily caused by unintended, leaky expression of the pIX gene present in the vector. In fact, when pIX leaky expression is avoided, vector expression can be sustained for up to 6 months, largely resolving the problem of high immunogenicity [25].

Despite the many advantages of AdVs mentioned above, there have been few reports of therapeutic knock‐in strategies using AdVs compared to those using AAV vectors [26]. Therefore, development of AdVs possessing high knock‐in efficiency will allow broader application in genome editing therapeutics. In plasmid‐based donor systems, the use of linearized donors or those with programmed end‐cleavage has been shown to markedly enhance knock‐in efficiency by facilitating nonhomologous end joining (NHEJ) or microhomology‐mediated end‐joining (MMEJ) pathways [27, 28, 29]. Similarly, while AAV genomes are primarily packaged as ssDNA, several studies have demonstrated that Cas9‐mediated cleavage of AAV donor terminals—which can occur following the formation of double‐stranded intermediates or by utilizing self‐complementary AAV (scAAV)—can further improve knock‐in rates in specific contexts [27]. However, the use of scAAV has also been associated with reduced knock‐in precision [30]. Because AdVs naturally possess a linear dsDNA genome, they are inherently structured to undergo programmed terminal cleavage without requiring prior second‐strand synthesis. While this structural characteristic suggests that intentional donor DNA truncation could be highly effective for AdV‐mediated knock‐in, this possibility remains to be fully investigated. Exploring this strategy may provide a significant breakthrough, positioning AdVs as a powerful tool for diverse genome editing therapies. This approach may be effective for AdVs because of their double‐stranded genomes, but this possibility has not been investigated.

We have developed AdVs expressing up to eight multiplex guide RNAs (gRNAs) [31, 32, 33, 34]. In this study, we constructed AdVs designed not only to express six multiplex gRNAs but also to carry a large donor DNA of 3.7 kb containing a normal exon 7 of the *Pah* gene. We then used these vectors with a knock‐in strategy to treat PKU model (*Pah* ^enu2^) mice. Cleaving the terminals of the donor DNA using a native Cas9 increased knock‐in efficiency by approximately twofold. When we intravenously co‐administered a donor DNA‐carrying AdV and an AdV expressing native Cas9 to newborn mice, blood phenylalanine levels were reduced by up to 30%. However, we also observed unintended rearrangements resulting from NHEJ. Therefore, although AdVs can be a useful platform for in vivo knock‐in, improvements are needed to increase knock‐in efficiency and to prevent native Cas9‐induced off‐target double‐strand breaks.

## 2 Materials and Methods

### 2.1 Cell Culture

Human 293 (ATCC CRL‐1573), HepG2 (ATCC HB‐8065), and Hepa1–6 (ATCC CRL‐1830) cell lines are derived from human embryonic kidney, human hepatocellular carcinoma, and C57L mouse BW7756 tumor cells, respectively. 293 cells were cultured in Dulbecco's modified Eagle's medium (DMEM; Kohjin Bio Co. Ltd., Saitama, Japan) supplemented with 10% fetal calf serum (FCS). 293 cells constitutively express adenoviral E1 genes and support the replication of E1‐substituted AdVs. HepG2 and Hepa1–6 cells were kept in high‐glucose DMEM (Kohjin Bio) supplemented with 10% FCS.

### 2.2 Construction of Cosmids Containing the AdV Genome Bearing Multiplex gRNA Units and Donor DNA

Multiplex (six) gRNA expression units targeting the mouse *Pah* gene and AdV were constructed in accordance with the Tetraplex‐guide Tandem method [33]. The head‐block reverse primer, ampHeadTE‐BxSs‐R, and the mid‐block forward primer, ampMidTA‐BxSs‐F, were used instead of ampl Head‐A R and ampl Mid‐A F [32]. The PCR products of head block and mid block were double‐digested with BstXI and BsaHI and cloned into the ClaI site in the E4 region of psAxp4wpcp‐g. The 3.7‐kb donor DNA including normal exon 7 was excised using SspI and Eco53kI and inserted into the SwaI site located adjacent to the six gRNA units. Multiplex (two) gRNA expression units targeting the AdV were constructed by the Tetraplex‐guide Tandem method [33], amplified by PCR, and cloned into the SgrDI site in the E1 region. The gRNA target sequences were 5′‐AACGGCGTCAATTGCATGCA‐3′ and 5′‐GCGTCAATTGCATGCAAGGTC‐3′ for Ax6g‐cut and 5′‐CAAATAAATGTTTCTGTCAA‐3′ and 5′‐CTTTATCATCCACATGCCTG‐3′ for Ax3DN‐cut. All the fragments were inserted in a reverse orientation.

### 2.3 Production of AdVs

AdVs were produced as previously described [31, 33]. Briefly, 293 cells were transfected with BstBI‐linearized cosmids containing the AdV genome. The next day, the cells were transferred to a 96‐well plate. The first viral stocks were obtained within 2 weeks (approximately 150 μL). Cells in 24‐well plates were infected with half of the first stock to obtain the second stock, and one‐tenth of the second stock was used for further stock amplification. AdVs used for mouse experiments were purified using a two‐step CsCl gradient as described previously [33]. AdVs were titrated using real‐time PCR to measure the viral genome copy numbers that successfully transduced infected HepG2 cells [35]. The relative viral titer (rVT) is normally equivalent to TCID_50_ and plaque‐forming units.

The AdVs expressing Cas9/Cas9 nickase under the control of the CB promoter, AxCBCas9/NC9, are available from the RIKEN BioResource Bank (https://dnaconda.riken.jp/search/RDB_clone/RDB19/RDB19290.html and https://dnaconda.riken.jp/search/RDB_clone/RDB19/RDB19291.html).

### 2.4 In Vitro Adv Infection and Conventional PCR

Hepa1–6 cells in 24‐well cell culture plates were infected with AdVs at the indicated multiplicity of infection (MOI) and incubated for 3 days postinfection in DMEM supplemented with 5% FCS. Total cellular DNAs were prepared and amplified by PCR using Tks Gflex DNA polymerase (Takara Bio Inc., Kusatsu, Japan) and primers: LaF, 5′‐ACAATATGAACCAACAAGTACCTCC‐3′; 4nR, 5′‐CGGAAGGCTAGGCCTCCTAG‐3′; 4nF, 5′‐GAGATTTCCTAGGAGGCCTA‐3′; RaR, 5′‐CACAGGACAGCCTCATAGCTTGTCC‐3′; sRaR, 5′‐TCATTTACTCTTCCATGATCCCAGC‐3′; and R‐AdR, 5′‐AATTTGCGCACCATGGACCAGCAGGTGGT‐3′. Images of DNA agarose gels after electrophoresis were recorded using a Printgraph Classic gel documentation system (ATTO Corp., Tokyo, Japan) and were analyzed using Image J 1.52a software [33].

### 2.5 AdV Infection of Mice

Animal experiments were approved by the Institutional Committee for Animal Experiments of Juntendo University (Approval No. 2021211). All procedures were performed in accordance with the ARRIVE guidelines and relevant regulations to minimize animal suffering. Animals were maintained in a temperature‐controlled environment (24°C ± 2°C) with a 12‐h light/dark cycle. They were housed in groups of four to five per cage and provided with standard chow (CLEA Japan Inc., Tokyo, Japan) and sterile water ad libitum. PAH‐deficient C57BL/6‐*Pah* ^enu2^ mice (*Pah* ^enu2^ mice) [36] were used, which are homozygous for the *Pah* ^enu2^ mutation described in the original BTBR‐*Pah* ^enu2^ strain [37, 38]. A total of eight male *Pah* ^enu2^ newborn mice were used in this study. For the treatment group, five newborn mice were randomly selected from the eight. These five mice received a primary intervention on postnatal day 1 via a 50‐μL intravenous injection of AdV into the facial vein using a 27G needle. Subsequently, the same five mice received a secondary challenge at 8 weeks of age, consisting of 150 μL of AdV administered via the tail vein. The remaining three mice served as untreated controls. Serum phenylalanine levels were determined at 5 and 13 weeks of age using a Phenylalanine Assay Kit (ab83376) from Abcam (Cambridge, UK). Mice were euthanized by cervical dislocation on the indicated days to harvest the liver tissue. Total DNAs were prepared from liver cells and amplified by PCR as described for the in vitro analysis. The T7EI assay was performed as described previously [33] using PCR fragments amplified by primers LaF and sRaR, except that the knock‐in fragments were removed from the sample by digesting the PCR fragments with StuI. To minimize bias, serum phenylalanine assay was performed by investigators blinded to the experimental groups. Animals were monitored daily for health and behavior; no animals reached humane endpoints requiring early euthanasia during the study. No animals or data points were excluded from the analysis.

### 2.6 Statistical Analysis

Data for serum phenylalanine concentrations were expressed as boxplots with individual plots. To compare the differences between the control and AdV‐treated groups, a two‐tailed Welch's *t*‐test was applied to account for unequal sample sizes. All calculations were executed using Microsoft Excel 2019.

## 3 Results

### 3.1 Construction of Knock‐in AdVs

**FIGURE 1:**
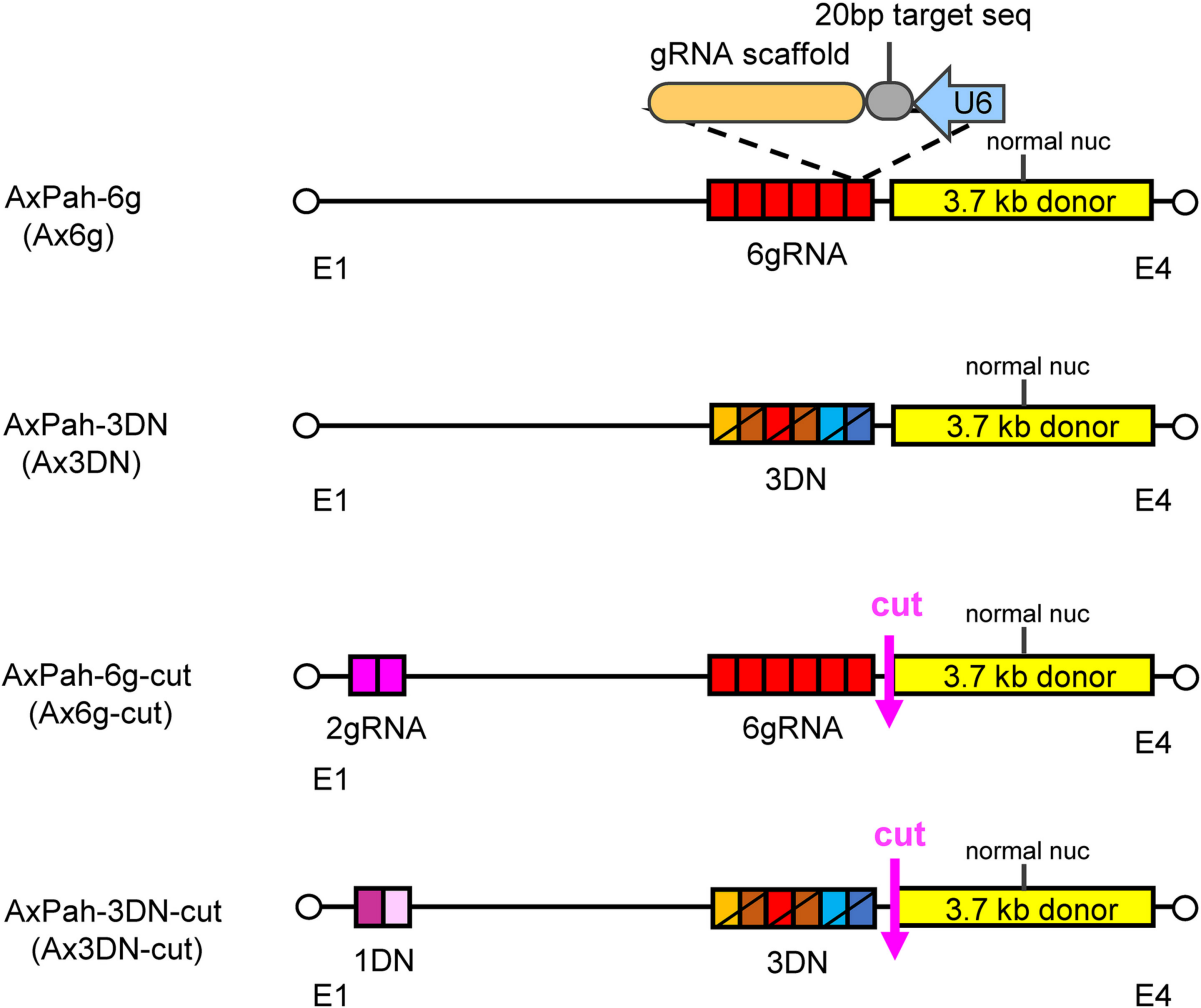
Structures of the knock‐in AdVs with donor DNA and multiplex gRNA expression units. All four AdVs carry a donor DNA at the E4 site of a 3.7‐kb genomic DNA fragment of the mouse *Pah* gene with exon 7 in the center. Also at the E4 site, AxPah‐6g (Ax6g) and AxPah‐3DN (Ax3DN) possess six reverse‐oriented expression units targeting exon 7 of *Pah*. Ax6g expresses six identical gRNA d units (red boxes, Figure 2). Ax3DN expresses two identical gRNA a units (brown boxes) and four other gRNAs, b to e. AxPah‐6g‐cut (Ax6g‐cut) and AxPah‐3DN‐cut (Ax3DN‐cut) carry two additional gRNA expression units at the E1 site of Ax6g and Ax3DN, respectively. The two gRNAs expressed from Ax6g‐cut (target sequences, 5′‐GAACGGCGTCAATTGCATGCA‐3′ and 5′‐GCGTCAATTGCATGCAAGGTC‐3′) are designed to cut the left terminus of the donor DNA via Cas9 cleavage. The gRNAs from Ax3DN‐cut (target sequences, 5′‐GCAAATAAATGTTTCTGTCAA‐3′ and 5′‐GCTTTATCATCCACATGCCTG‐3′) are designed to cut the left terminus of the donor DNA by Cas9n‐mediated double‐nicking. All gRNAs described above are expressed using a U6 promoter. Ax6g and Ax6g‐cut were used in combination with AxCBCas9, while Ax3DN and Ax3DN‐cut were used with AxCBNC9.

**FIGURE 2:**
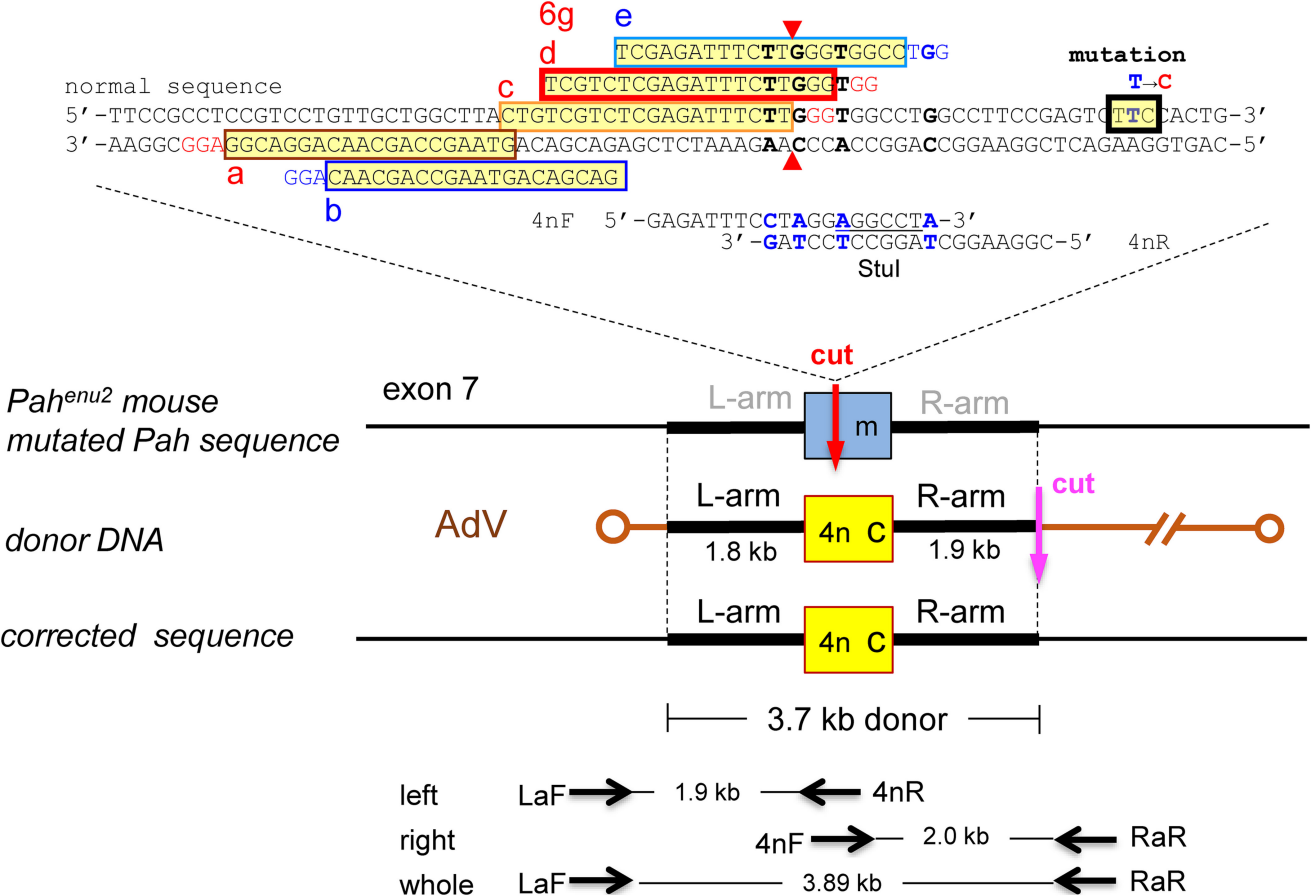
Strategy for mouse *Pah* exon knock‐in by HDR. The 3.7‐kb donor DNA in the knock‐in AdVs is indicated by a thick black line. The gRNA‐targeted sites in exon 7 are flanked by 1.8 kb left (L) and 1.9 kb right (R) homology arms. The T to C mutation in the *Pah* ^enu2^ mouse is shown at the right‐hand end. The four nucleotides shown in blue below the sequence represent the bases that have been altered in the donor DNA. Three of these four nucleotides (C, A, A) overlap with the 20‐nucleotide target sequence of the therapeutic gRNAs, c, d, and e, which prevents these gRNAs from cleaving the donor DNA. One of the artificial nucleotides (the second A) creates a StuI cleavage sequence (underlined) without changing the amino acid sequence. The presence of the StuI site in the successfully knocked‐in cellular DNA facilitates its detection. The five gRNA target sequences are shown as a to e. Sequence d is the target for the gRNA expressed from the E4 site of Ax6g and Ax6g‐cut. Three sets of double‐nicking gRNA pairs (a/c, a/d, and b/e) are expressed from the E4 site of Ax3DN and Ax3DN‐cut. The locations of PCR primers (arrows) and the lengths of PCR fragments are indicated in the bottom section.

We first constructed AxPah‐6g and AxPah‐3DN vectors (hereafter referred to as Ax6g and Ax3DN, respectively; DN stands for double‐nicking; Figure 1). Both vectors share a large common 3.7 kb donor DNA that contains normal *Pah* exon 7 in its center. The Ax6g vector contains six copies of identical gRNA expression units (red boxes). This vector was used for co‐infection together with the Cas9 expression vector, AxCBCas9. The Ax3DN vector also contains six gRNA expression units, although they are composed of three pairs of double‐nicking expression units. These six units include two identical gRNA a units (two brown boxes) and four different expression units for gRNA b to gRNA e (the sequences of all gRNAs are presented in Figure 2, upper panel). One of these units (red box) is identical to the six gRNA units in the Ax6g vector. The Ax3DN vector was used for co‐infection together with the Cas9 nickase (Cas9n) expression vector, AxCBNC9. The expression of the five different gRNAs aims to achieve efficient and safe cleavage through three sets of double‐nicking.

Figure 2 shows the location of the disease‐causing mutation in exon 7 of the *Pah* gene in the *Pah* ^enu2^ mouse model (upper panel, right, T to C) and the sequences of the five gRNAs used (upper panel, center, gRNAs a to e). To obtain the highest cleavage efficiency by native Cas9, each of the five gRNAs was assayed for efficiency in vitro, and gRNA d showed the highest efficiency (Figure S1). Consequently, gRNA d was chosen for the Ax6g vector, which contains six identical expression units of gRNA d (Figure 1). The Ax3DN vector contains all five gRNAs, which produce three double‐nicking cleavages by gRNAs c, d, and e on the top strand in cooperation with gRNAs a and b on the bottom strand, expecting an additive effect for high cleavage efficiency (Figure 1).

The donor DNA sequences contain four artificially altered nucleotides, which do not change the amino acid sequence (Figure 2), (upper panel, center, four blue and bold nucleotides in the overlapping region of the 4nF and 4nR primers containing the StuI site). These alterations, located approximately 20 bases upstream of the causative mutated nucleotide, are absent from wild‐type genome sequences. These alterations serve two functions: they prevent the donor DNA from being cleaved by native Cas9 or double‐nicking by Cas9n, and they create a unique StuI restriction site (underlined), which is used for distinguishing the donor DNA of the vector from cellular genomic DNA. These alterations enabled the design of PCR primers that specifically distinguish the successfully knocked‐in genome from the unchanged target genome.

To generate the Ax6g and Ax3DN vectors, the AdV genome was excised from a cosmid and subsequently transfected into 293 cells. To examine a lack of deletion of gRNA units due to homologous recombination, total cellular DNA from second viral stocks was prepared and digested with SmaI. The 6.8‐kb bands of Ax3DN and Ax6g, which contain the six gRNA unit sequence (Figure S2A, labeled as 6g), were not much less intense than the 6.4‐kb band derived from the AdV backbone beneath the 6.8‐kb band (labeled as A), indicating that the six multiple or multiplex units were mostly maintained without deletion. Even in the third to fifth viral stocks that were further amplified, the intensity of the 6.8‐kb bands was at least 70% of that from the band derived from the AdV backbone (Figures S2B and S2C). This reduction is probably due to the deletion of gRNA units via homologous recombination because ladder bands corresponding to four, three, and two gRNA units were also detected (Figures S2B and S2C, evident in 4th stock lanes). These results indicate that, although the six expression units are either completely identical or differ only by 20 bases, they were stably maintained, and, consequently, over 70% of the AdVs retained an intact array of all six units without deletion.

### 3.2 Examination of In Vitro Knock‐in Efficiency Using AdVs

**FIGURE 3:**
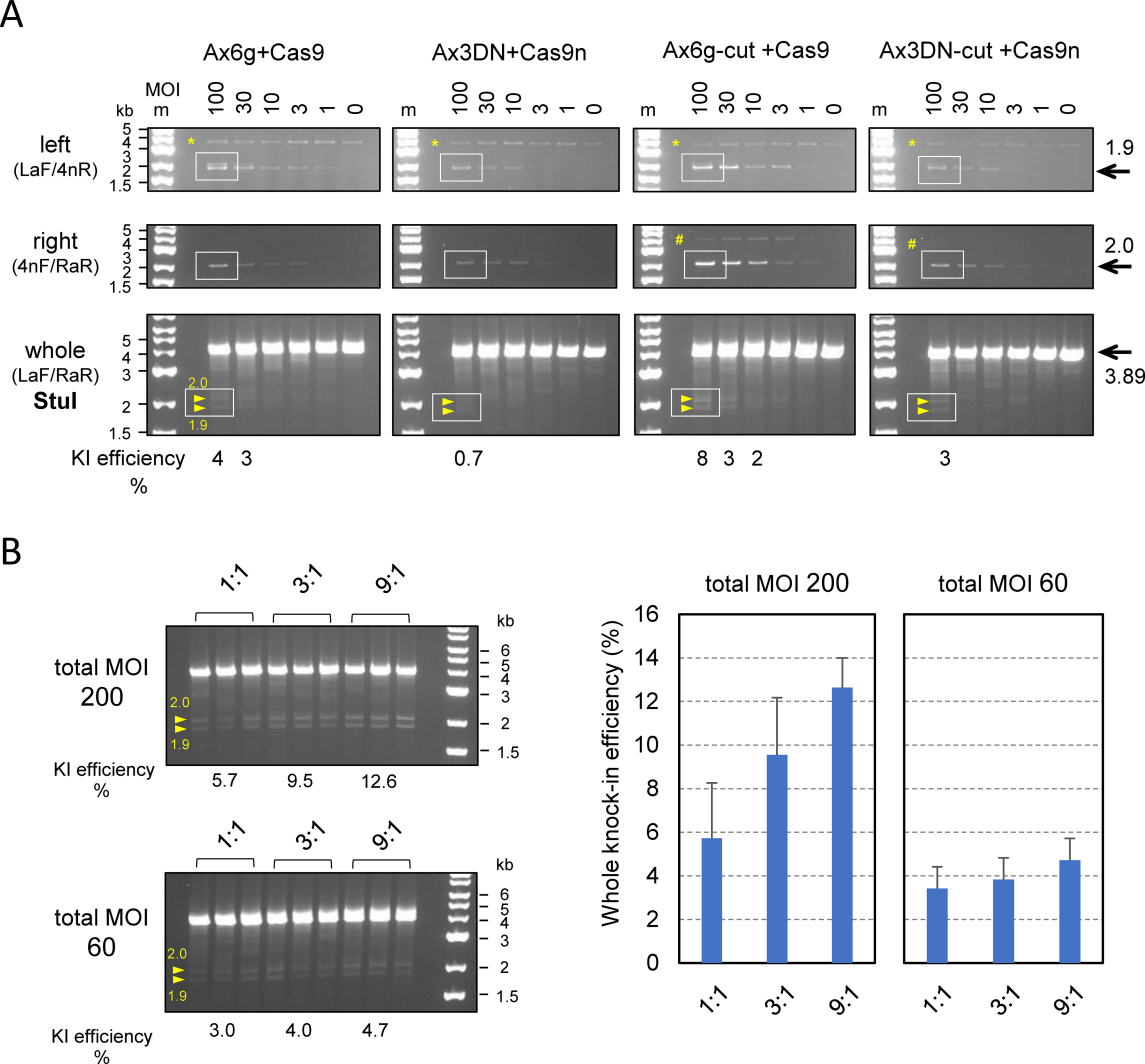
In vitro knock‐in via HDR at exon 7 of the mouse *Pah* gene using knock‐in AdVs containing a 3.7‐kb donor DNA. (A) Knock‐in efficiency with different AdVs. Hepa1–6 cells were infected with the knock‐in AdVs (Ax6g, Ax3DN, Ax6g‐cut, and Ax3DN‐cut) and Cas9/Cas9 nickase‐expressing AdVs (AxCBCas9/NC9) at MOIs of 100, 30, 10, 3, 1, and 0. Total cellular DNA was extracted 3 days postinfection and subjected to 30 cycles of PCR amplification using primers shown in Figure 2. The 4nR primer and the 4nF primer each contain a four‐nucleotide donor DNA‐specific artificial sequence, and do not pair with the native mouse genomic DNA. Therefore, this PCR specifically detects only the knocked‐in genomic DNA sequence, and not the pretreated mouse genomic sequence. The asterisk (*) in the first row indicates a nonspecific PCR band derived from the mouse genome. The approximately 3.9 kb band marked with a hash (#) may originate from NHEJ‐mediated knock‐in, which is explained as NHEJ 1 in Figure 5A. Knock‐in efficiency was determined by digesting the 3.89‐kb PCR fragment with StuI (as shown in Figure 2) and subsequent analysis by gel electrophoresis. The 2.0‐ and 1.9‐kb bands were quantified, and the efficiency was calculated as the ratio of these bands to the total DNA in the same lane. (B) Enhancement of knock‐in efficiency by modulating the vector ratio. Hepa1–6 cells were infected with Ag6g and the Cas9 expressing vector at ratios of 1:1, 3:1, and 9:1, with total MOIs of 200 and 60. The knock‐in efficiency was calculated by digesting the 3.9‐kb PCR fragment with StuI as described in A.

The knock‐in efficiency of the AdVs was first examined using mouse hepatoma‐derived Hepa1–6 cells as an in vitro knock‐in model of hepatocytes. While Hepa1–6 cells do not have a mutant *Pah* gene in the *Pah* ^enu2^ PKU mouse model, the knock‐in efficiency can be determined by detecting a specific four‐base alteration in the donor DNA introduced into the knocked‐in cells. Hepa1–6 cells were first infected with the Ax6g vector expressing high levels of gRNA d only (Figure 2, upper, gRNA d, the sequences are boxed in red) at an MOI of 0, 1, 3, 10, 30, and 100 together with AxCBCas9 at the same MOI. After 3 days, the total cellular DNA was extracted, and the knocked‐in L‐arm region was amplified by PCR using primers LaF and 4nR (Figure 2, lower, L‐arm in “corrected sequence” and “left” primers of LaF and 4nR; note that LaF primer is located outside of the L‐arm region). Similarly, the knocked‐in R‐arm region was specifically detected using the 4nF primer and the RaR primer (R‐arm in “corrected sequence” and “right” primers of 4nF and RaR). The knock‐ins of both left and right regions were observed using Ax6g together with AxCBCas9 (Figure 3A, first column, first and second rows; the bands at MOI 100 are boxed). Interestingly, the knock‐in efficiencies of the L‐arm and R‐arm regions were not the same. The knock‐in appeared to be more difficult in the R‐arm region because the band of the left region was more intense than that of the right region (both bands are boxed; see also Figure S4).

However, it remained unclear whether both regions were knocked‐in simultaneously. Therefore, the 3.89‐kb PCR fragment covering the whole region was amplified with primers LaF and RaR, which are located outside of the donor DNA (Figure 2, lower, bottom; labeled “whole”), and the resultant fragment was digested with StuI. This knocked‐in fragment contains not only the four‐nucleotide alteration but also an overlapping StuI site, while the wild‐type fragment does not. StuI restriction of the entire knock‐in fragment produces 2.0 and 1.9 kb fragments. The knock‐in efficiency for the entire fragment was determined to be 4% using Ax6g at MOI 100 (Figure 3A, column Ax6g+Cas9, row whole/StuI). We also evaluated whether knock‐in occurs via double‐nicking cleavage of genomic DNA by co‐infection of Ax3DN with the Cas9 nickase expression vector, AxCBNC9 (Figure 3A, Ax3DN+Cas9n). We clearly observed knock‐in for the L‐arm and R‐arm regions using Cas9n. However, the knock‐in efficiency for the entire fragment for Ax3DN+Cas9n was only 0.7%, which was considerably lower than that of Ax6g+Cas9. The low efficiency was corroborated by measuring the knock‐in efficiencies of the L‐arm and R‐arm regions at MOI 100 (Figure 3B).

### 3.3 Cleaving Donor DNA From the AdV Increases Knock‐in Efficiency

To examine whether cleavage at the terminus of the donor DNA in the AdV genome enhances knock‐in efficiency, we constructed two AdV vectors, Ax6g‐cut and Ax3DN‐cut. These vectors were derived from Ax6g and Ax3DN, respectively, by adding two gRNA expression units into the E1 cloning site (Figure 1). In Ax6g‐cut, native Cas9 mediates double‐strand cleavage at two adjacent sites in the left terminus of the donor DNA, while in Ax3DN‐cut, Cas9n induces double‐nicks in the left terminus of the donor DNA. During construction of Ax6g‐cut and Ax3DN‐cut, the stability of the six gRNA units present in the E4 cloning site did not differ from Ax6g and Ax3DN (Figures S3B, S3D, and S3E). In contrast, the band derived from the E1 site, containing two gRNAs, did not show any deletion (Figure S3).

When Hepa1–6 cells were co‐infected with Ax6g‐cut plus AxCBCas9 at MOI 100, knock‐in bands of the L‐arm and especially the R‐arm regions were more intense than those with Ax6g and AxCBCas9 (Figure 3A, compare the intensity of the bands in the boxes of the first and third columns, first and second rows; Figure S4, left and right, compare Ax6g+Cas9 and Ax6g‐cut+Cas9). Remarkably, the knock‐in efficiency of the whole region using Ax6g‐cut plus AxCBCas9 reached 8% at MOI 100, representing a twofold increase over the use of Ax6g plus AxCBCas9 (first and third columns, third row, KI efficiency 4% and 8%, respectively). These results indicate that the knock‐in efficiency was significantly increased by cleavage of the donor DNA terminus in the AdV genome. Moreover, the knock‐in efficiency of Ax3DN‐cut plus AxCBNC9 reached 3%, which was almost fourfold higher than that of Ax3DN plus AxCBNC9 (second and fourth columns, third row, KI efficiency 0.7% and 3%, respectively). However, the efficiency was still less than half that of Ax6g‐cut plus AxCBCas9. Therefore, subsequent studies were performed using Ax6g‐cut together with AxCBCas9.

To examine the effect of ratios of Ax6g‐cut to AxCBCas9, Hepa1–6 cells were infected with these AdVs at a total MOI of 200 with the ratios of 1:1, 3:1, and 9:1. The knock‐in efficiencies were 5.7%, 9.5%, and 12.6%, respectively (Figure 3B, upper photo and left graph), indicating that a greater amount of Ax6g‐cut gave a higher knock‐in efficiency. At a total MOI of 60 (i.e., MOIs of 54 and 6 for Ax6g‐cut and AxCBCas9, respectively), the 9:1 ratio was the most efficient (lower photo and right graph), and this effect was more pronounced at a higher total MOI.

Tracking of insertions, deletions, and end‐joining repair (TIDER) analysis [39, 40] is a modified version of TIDE analysis that estimates the frequency of targeted small nucleotide changes introduced by CRISPR in combination with HDR using a donor template. Knock‐in efficiency determined using TIDER analysis produced comparable results to those obtained via PCR analysis combined with StuI digestion (Figure S5). Furthermore, the addition of NHEJ inhibitors (vanillin and SCR7) or HDR promoters (L755507) at the indicated concentrations slightly increased knock‐in efficiency in Hepa1–6 cells (Figure S6).

### 3.4 Examination of In Vivo Knock‐in Treatment of *Pah* ^enu2^ Mice

**FIGURE 4:**
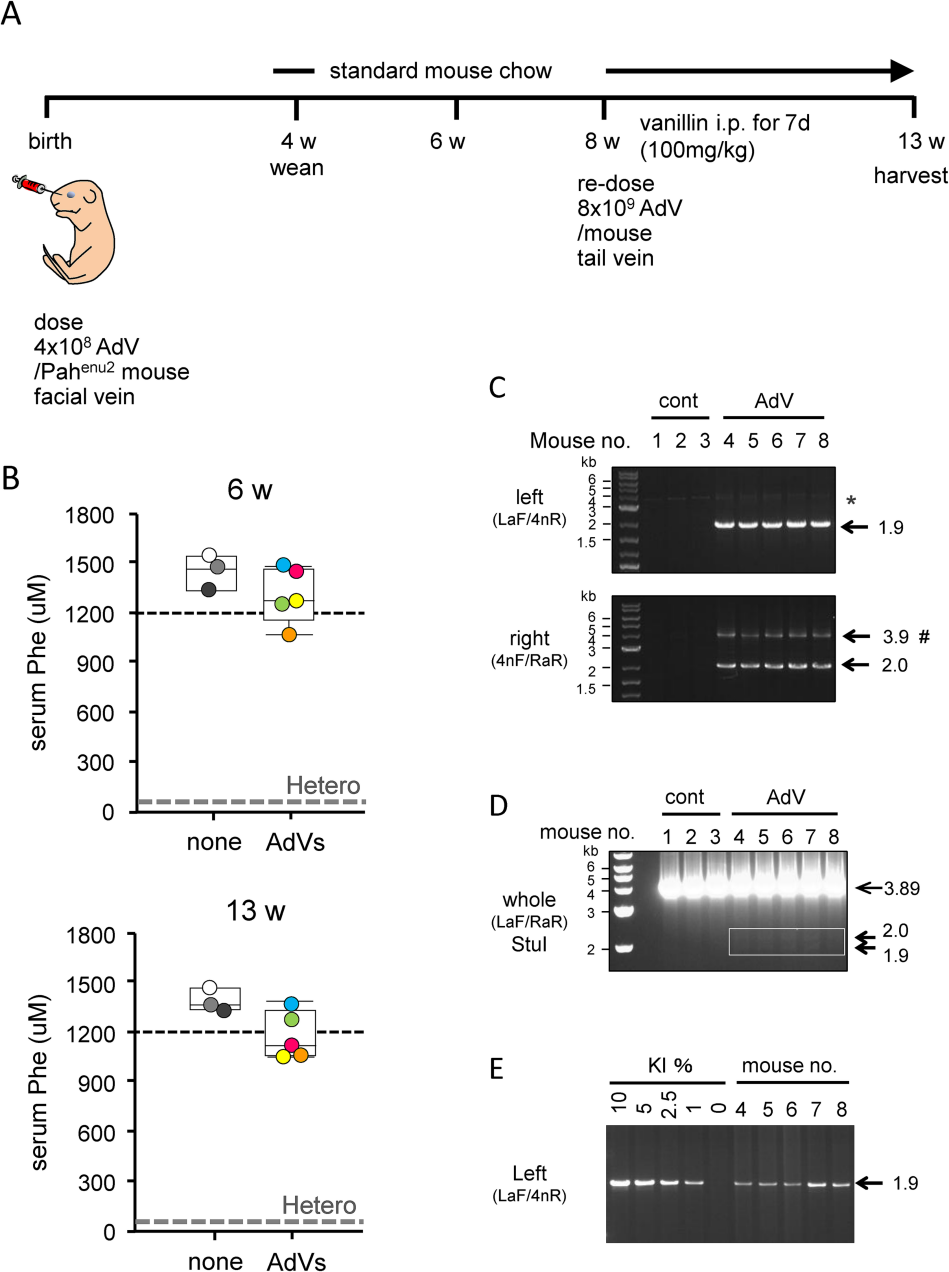
In vivo knock‐in of neonatal mouse liver cells administered with Ax6g‐cut and AxCBCas9. (A) Time course of the study. Five neonatal *Pah* ^enu2^ mice were initially administered with AdVs at 4 × 10^8^ rVT/mouse via the facial vein, and re‐dosed at 8 weeks with 8 × 10^9^ rVT/mouse. The ratio of Ag6g‐cut to AxCBCas9 was 3:1. Daily administration of 100 mg/kg vanillin was performed for 7 days following the re‐dose. (B) Effect of AdV administration on serum phenylalanine levels. Serum phenylalanine (Phe) concentrations were measured in control (none, *n* = 3, mouse nos. 1–3) and AdV‐treated mice (AdVs, *n* = 5, mouse nos. 4–8) at 6 weeks (top) and 13 weeks (bottom) of age. Data are presented as boxplots with individual data points (mean ± SD at 6 weeks: control = 1464 ± 105, AdVs = 1319 ± 168; at 13 weeks: control = 1398 ± 67, AdVs = 1188 ± 144). The black dashed line at 1200 μM represents the threshold separating classical PKU from mild cases, while the gray dashed line indicates the reference Phe level for heterozygous mice (Hetero). Statistical analyses were performed using a two‐tailed Welch's *t*‐test (*p* = 0.18 at 6 weeks; *p* = 0.03 at 13 weeks). (C) Analysis of liver cells by PCR. Cellular DNA was prepared from the liver at 13 weeks and subjected to conventional PCR. Left and right region knock‐in events were detected as 1.9 and 2.0 kb bands (arrows), respectively, after 35 cycles of PCR. The asterisk (*) in the first row indicates a nonspecific PCR band derived from the mouse genome. The 3.9‐kb bands marked with a hash (#) may originate from NHEJ‐mediated knock‐in, which is explained as NHEJ 1 in Figure 5A. (D) Detection of simultaneous left and right region knock‐in. Cellular DNA was amplified by 35 cycles of PCR using primers LaF and RaR and subsequently digested by StuI. In addition to the 3.89‐kb PCR fragment, faint 2.0 and 1.9 kb bands were detected, indicating that simultaneous knock‐in had indeed occurred. (E) Calculation of knock‐in efficiency for the left region. Cellular DNA was subjected to 30 cycles of PCR using primers LaF and 4nR. The PCR products from treated mice were compared with those from a control DNA mixed with a known percentage of knocked‐in fragments. The knock‐in efficiency of the left region was approximately 1% in treated mice.

To examine the efficiency of in vivo hepatocyte knock‐in, five newborn mice were treated with Ax6g‐cut and AxCBCas9. These AdVs were administered intravenously at 3 × 10^8^ rVT and 1 × 10^8^ rVT, respectively (Figure 4A). Serum phenylalanine levels were measured 6 weeks after AdV administration. In one mouse, the serum phenylalanine level was decreased below 1200 μM (Figure 4B, upper). Classical PKU is categorized by phenylalanine levels above 1200 μM, and patients with levels below this are classified as mild cases. The effect observed in this mouse is therefore possibly significant. At 8 weeks, the mice received a second intravenous administration of Ax6g‐cut and AxCBCas9 at 6 × 10^9^ rVT and 2 × 10^9^ rVT, respectively, with additional daily intraperitoneal administration of vanillin (100 mg/kg) for 7 days. Five weeks after the second administration, serum phenylalanine concentrations in three out of the five mice were decreased below 1200 μM by up to approximately 30% (Figure 4B, bottom), confirming that this vector system was effective. Remarkably, this result also showed that the second administration in this system enhanced the therapeutic effect, although re‐administration of AdVs is generally not effective (see Discussion).

At 13 weeks, the mice were euthanized and cellular DNA from the liver was prepared. The knock‐in efficiencies were examined separately for the L‐arm region using primers LaF and 4nR and for the R‐arm region using primers 4nF and RaR, as shown in Figure 2. PCR products of 1.9 and 2.0 kb, respectively, were detected in all five treated mice as expected (Figure 4C). To calculate the knock‐in efficiency of the whole region, the 3.89‐kb PCR product amplified using LaF and RaR was digested with StuI, as in the in vitro study. Very faint 2.0 and 1.9 kb bands were detected below the 3.89‐kb band (Figure 4D). This indicates that the entire knock‐in indeed occurred in hepatocytes, although the efficiency was too low to quantify in this assay. The knock‐in efficiency was also below the level that could be estimated by TIDER analysis (Figure S7). We therefore calculated the knock‐in efficiency of the L‐arm region by comparing the products from a 30‐cycle PCR with those of the control, which contained a known percentage of the knock‐in fragment. This analysis showed the knock‐in efficiency of the L‐arm region in the treated mice to be approximately 1% (Figure 4E).

**FIGURE 5:**
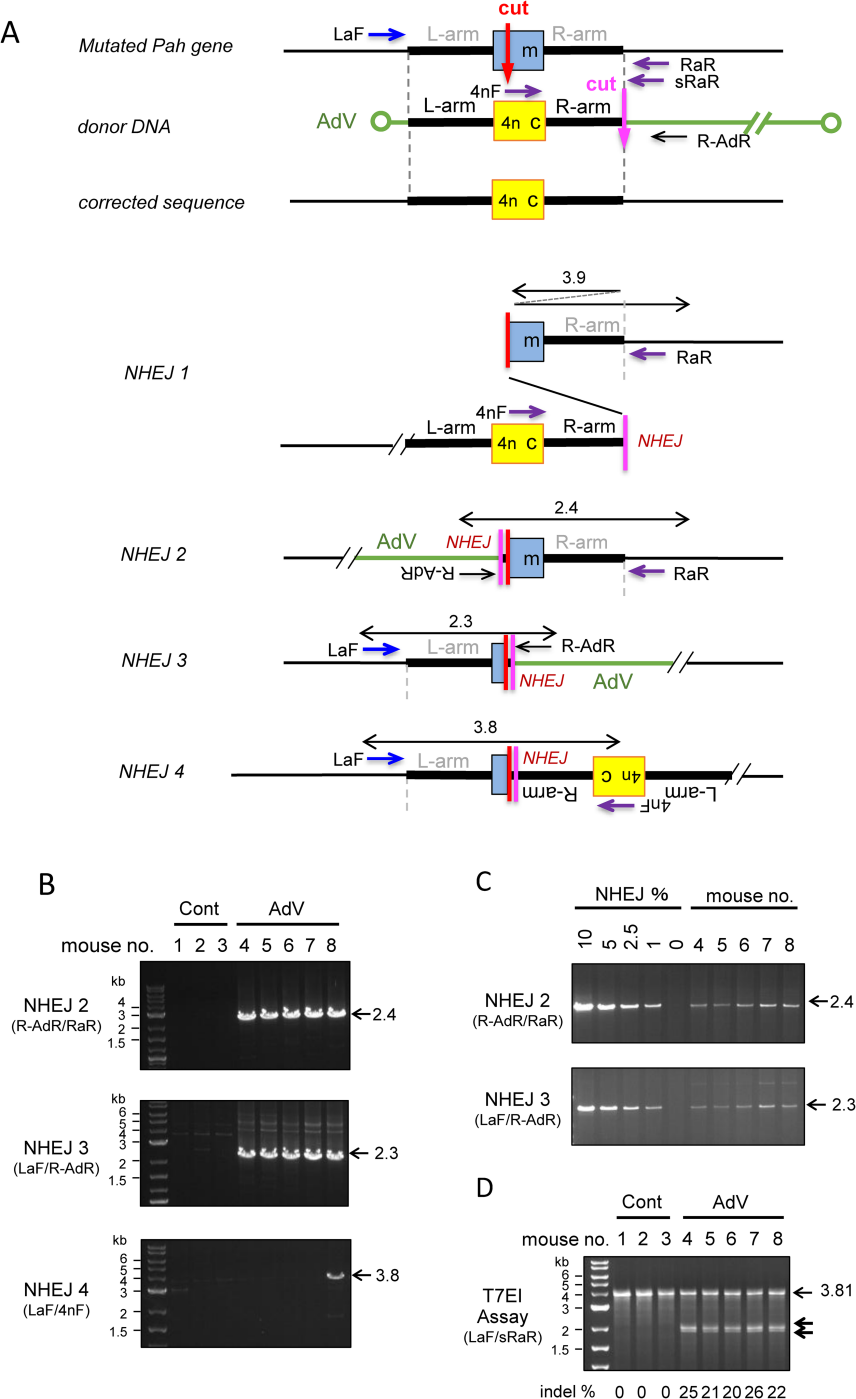
Detection of unintended knock‐in events, including NHEJ and indels, in liver cells of AdV‐treated mice. (A) Schematic diagram of possible NHEJ events. NHEJ 1 was detected by PCR as an approximately 3.9 kb fragment (Figure 4C, second row). NHEJ 2, 3, and 4 were detected by PCR using primer sets R‐AdR/RaR, LaF/R‐AdR, and LaF/4nF. (B) NHEJ detected by PCR in liver cells of AdV‐treated mice. To detect NHEJ 2 to NHEJ 4, cellular DNAs from liver of control (nos. 1–3) and AdV‐treated mice (nos. 4–8) were amplified by PCR using primer sets R‐AdR/RaR, LaF/R‐AdR, and LaF/4nF to generate 2.4, 2.3, and 3.8 kb fragments, respectively. (C) Calculation of NHEJ efficiency. Cellular DNA was subjected to 30 cycles of PCR using primer sets R‐AdR/RaR and LaF/R‐AdR to detect NHEJ 2 and NHEJ 3. The PCR products from treated mice were compared with those from a control DNA mixed with a known percentage of the corresponding fragments. The efficiency of NHEJ 2 and NHEJ 3 in treated mice was approximately 1%. (D) Indel analysis at the gRNA sites. Cellular DNAs from the liver of control mice (nos. 1–3) and AdV‐treated mice (nos. 4–8) were amplified by PCR using primer set LaF/sRaR and digested by StuI. The 3.81‐kb band was purified by gel extraction and the 3.81‐kb fragments that did not contain the intended knock‐in sequence were subjected to T7EI assays. The indel percentage was calculated by measuring the 1.87‐kb and 1.94‐kb fragments relative to the 3.81‐kb band in the same lane.

Four types of NHEJ were detected in hepatocytes by PCR (Figure 5). NHEJ 1 was detected in all five mice as a 3.9‐kb PCR fragment above the 2.0‐kb PCR fragment (Figure 4C, lower, marked as #). NHEJ 2 and 3 were also detected in all mice as PCR fragments of approximately 2.4 and 2.3 kb, respectively, using the primer sets R‐AdR/RaR and LaF/R‐AdR (Figure 5B). However, NHEJ 4 was only detected in mouse 8. The efficiency of NHEJ 2 and 3 was calculated by comparing the 30‐cycle PCR products with those of the control, which contained a known proportion of knock‐in fragments. The efficiency was approximately 1% (Figure 5C). The structures of these NHEJ types are described and explained in the Discussion section.

The T7E1 assay determined the formation of short insertions/deletions (indels) at the knock‐in target site to have an efficiency of approximately 20% to 26% (Figure 5D). This indel efficiency correlated well with values determined by TIDER [39, 40], although this analysis showed a broader range of 28% to 42% (Figure S7). These results indicate that it is crucial to increase HDR‐mediated knock‐in efficiency and that indel formation should be reduced to improve this AdV knock‐in system.

## 4 Discussion

AAV vectors are most frequently utilized for knock‐in approaches in genome‐editing studies. However, AAV vectors can only carry a maximum of 4.7 kb of foreign DNA and, consequently, short homology arms, typically under 0.8 kb, are often employed [26]. The knock‐in efficiency mediated by HDR generally improves as the length of homology arms in the donor DNA increases [41]. AdVs, which can carry up to 8 kb of DNA, can potentially obtain high knock‐in efficiency by using much larger homology arms.

In this study, we took advantage of the large packaging capacity of AdVs to develop vectors carrying not only a 3.7‐kb donor DNA but also six multiplex gRNA expression units. We also demonstrated that the knock‐in efficiency was enhanced by cutting the donor DNA at its junction with the AdV vector backbone. When Hepa1–6 cells were co‐infected with the most efficient knock‐in AdV (Ax6g‐cut) and a Cas9‐expressing AdV, the HDR‐mediated knock‐in efficiency reached up to 8% in vitro. Because of these improvements, we obtained an approximately 30% reduction in blood phenylalanine concentration in vivo. These findings demonstrate that AdVs can be effective knock‐in vectors for genome‐editing therapy. However, their knock‐in efficiency in liver cells was still low at less than 1%. Also, unintended NHEJ events were detected at the cut site of the donor DNA (Figure 5A, NHEJ‐1). Therefore, the knock‐in system presented in this study requires improvements, which are probably achievable because the large capacity of AdVs allows for necessary modifications.

We here showed that the second administration was effective in this vector system (Figure 4B, lower). This is interesting because it is generally believed that second administration of AdVs is not effective due to severe immune response and neutralizing antibody [23, 24]. One possibility is that knock‐in quickly begins before the immunological rejection of the second administered AdV occurred. If its efficiency is improved, AdV could allow second administration in knock‐in therapies. Secondly, we used neonatal mice whose immune system is immature. Knock‐in therapy is desired specially for infants or children because AAV vectors can treat only adults since the vector genome is diluted by the growth of the child. Thirdly, we used an AdV system that avoids leak expression of the viral pIX gene, which is a major cause of the immune response of AdVs [25]. Effective second administration could be achieved because all of these conditions were fulfilled.

Current knock‐in therapies using genome‐editing technology mostly use native Cas9. However, the recognition specificity of native Cas9 is not high, and often results in off‐target cleavage, a major concern for the clinical application of genome‐editing therapies. A possible solution to this problem is the double‐nicking strategy, which employs Cas9n together with two adjacent gRNAs to produce a double‐strand cleavage. Using this approach, off‐target activity is reduced by up to 1/1500 compared with native Cas9 in transfection experiments [42]. Moreover, we showed that off‐target effects were below the limit of detection in the study using AdVs [33, 34]. Based on these findings, we applied Cas9n‐mediated double‐nicking cleavage to generate a double‐strand break at the knock‐in site to try to develop a safer knock‐in system. However, unexpectedly, the knock‐in efficiency using Cas9n‐mediated double‐nicking cleavage was much lower than that with native Cas9 cleavage. While the difference in DNA end structures (blunt ends vs. staggered ends) may influence the repair process, the efficiency of the double‐nicking strategy is also known to be highly sensitive to the orientation and spacing of the gRNA pairs. Since we selected gRNAs primarily based on their proximity to the target site, further optimization of these spatial parameters may be required to improve the performance of the Cas9n system. Therefore, while AdV‐mediated delivery of Cas9n remains a potential option for reducing off‐target effects, its efficiency relative to native Cas9 in this specific context requires more detailed investigation.

Cleavage of the donor DNA from the AdV genome resulted in an approximately twofold increase in knock‐in efficiency for native Cas9 (4% to 8%), and an approximately fourfold increase for Cas9n (0.7% to 3%). This finding aligns with several studies. For example, in experiments using plasmids, excising the donor DNA with native Cas9 by introducing gRNA target sequences on both ends of the donor DNA increased HDR efficiency by twofold to fivefold compared with using the uncleaved circular plasmid donor [29]. Similarly, although AAV possesses a single‐stranded genome, it generates double‐stranded intermediates during its life cycle. Strategies such as homology‐independent targeted integration (HITI) have been employed to cleave AAV donor DNA using guide RNAs, which have been shown to significantly enhance knock‐in efficiency [27]. Consistently, we demonstrated that for the AdV system, cleaving the donor DNA from the vector also enhanced knock‐in efficiency. Notably, since AdV possesses a naturally double‐stranded genome, it bypasses the requirement for second‐strand synthesis inherent to AAV, potentially facilitating a more streamlined and efficient integration process. As already described, we observed that when the cell genome was cleaved at the knock‐in target site using the double‐nicking strategy of Cas9n, the knock‐in efficiency was unexpectedly low, nearly one‐sixth of that using native Cas9 (Figure 2, middle section, first line, cut indicated in red; Figure 3A, second and first columns, 0.7% and 4%, respectively). However, interestingly, when the AdV genome was cleaved at the donor DNA terminus using the double‐nicking strategy of Cas9n, the knock‐in efficiency remarkably increased by more than fourfold compared with no cleavage (Figure 2, middle section, second line, cut indicated in pink; Figure 3A, second and fourth columns, 0.7% and 3%, respectively). The reason for these apparent differences is unclear but might be related to differences in the involvement of the former and latter in chromosomal and extra‐chromosomal events, respectively.

Although the knock‐in efficiency was increased by cutting the terminus of the donor DNA in the AdV genome (Figure 5A, upper panel, second line, pink vertical arrow with “cut” on the R‐arm), we found that donor cleavage terminus was efficiently connected to the terminus of the on‐target cleavage site in the cell genome (first line, red vertical arrow with “cut”) and undesirable NHEJ occurred. We observed four different NHEJ events caused by cutting the terminus of the donor DNA (Figure 5A, second panel, NHEJ 1 to NHEJ 4), and all of them disrupted the coding region. NHEJ 1 was detected in vivo in all five mice examined (Figure 4C, lower panel, 3.9 kb fragment, marked as #). This fragment was observed in vitro using not only Ax6g‐cut+Cas9 but also Ax3DN‐cut+Cas9n, indicating that this NHEJ event also occurred using double‐nicking cleavage (Figure 3A, middle two panels of the third and fourth rows, marked as #). The deduced structure showed that the NHEJ event caused an insertion accompanied with R‐arm duplication between the two cleavage sites (Figure 5A, NHEJ 1). In NHEJ 2, the AdV sequence to the right of the cleavage was inversely connected (upper panel, second row, pink vertical line adjacent to pale green AdV genome with inversed R‐AdR primer). In NHEJ 3, the AdV sequence was also connected despite the original orientation (third row). NHEJ 1 to NHEJ 3 were commonly observed in the five mice tested, indicating an extremely high frequency of these joining events (Figure 4C, right, 3.9 kb; Figure 5B, NHEJ 2 and NHEJ 3, mouse nos. 4–8). Indeed, the calculated knock‐in frequencies of NHEJ 1 to NHEJ 3 were nearly 1% (Figure 4E and Figure 5C, mouse nos. 4–8), which is similar to that of the intended knock‐in (Figure 4D). NHEJ 4 was detected in only one mouse (Figure 5B, NHEJ 4, mouse no. 8), indicating that its occurrence may not be frequent. The deduced structure can be an inverted duplication, where short R‐arm sequences were flanked with very large, inverted‐repeat sequences (the L‐arm sequence in this case) [43], which could be related to chromosomal rearrangements [44].

Because the frequency of HDR is lower than that of NHEJ in mammalian cells, blocking the NHEJ pathway and stimulating the HDR pathway are often applied to enhance HDR. Many small molecules can increase HDR efficiency through inhibition of key factors involved in NHEJ, such as DNA‐PK and ligase IV, and by stabilization of RAD51, a chromatin remodeling factor [9]. Furthermore, vanillin can significantly enhance therapeutic efficacy in PKU model mice treated with AAV vectors through increased HDR efficiency [13]. We therefore investigated vanillin (an inhibitor of DNA‐PK), SCR7 (an inhibitor of ligase IV), and L755505 (which promotes HDR through an unknown mechanism). We found that all of these molecules increased HDR efficiency in vitro, but the effect was only marginal. Subsequently, we tested vanillin in AdV‐treated mice, but the therapeutic results were not as great as those achieved with AAV vectors. This discrepancy may suggest that in vivo HDR efficiency might be influenced by a complex interplay of factors, including vector persistence, the strandedness of the viral genome (single‐stranded for AAV vs. double‐stranded for AdV), intracellular DNA processing, and the host immune response. To mitigate immunogenicity, we utilized neonatal mice and AdVs designed to avoid the leaky expression of the viral pIX gene, which is a major cause of the immune response of AdVs [25]. Nevertheless, further studies are needed to evaluate how transient immune activation might influence the repair pathway choice between HDR and NHEJ.

In conclusion, we have progressed the preclinical development of AdVs for genome‐editing therapy. To overcome the low knock‐in efficiency of AdVs, we developed AdVs carrying a donor DNA designed to not only have large homology arms but also to be cut at its terminus. Although our approach yielded a partial therapeutic effect in the PKU mouse model, we did not achieve robust efficacy. However, the AdV improvements described here offer a valuable basis for further development of AdVs to achieve effective vectors for future knock‐in therapy.

## Author Contributions

M.Y.: experimental investigation and data analysis. H.T.: cosmid construction and data analysis. M.N.: experimental investigation. R.F.: cosmid construction. Ta.O.: AdV production. S.O., To.O., and M.K.: mouse breeding. I.S.: conceptualization, data analysis, writing – review and editing. S.S.: writing – review and editing. T.N.: experimental investigation and data analysis, writing – original draft, and funding acquisition.

## Ethics Statement

Animal studies are reported in accordance with the ARRIVE guidelines. All experimental protocols were approved by the Institutional Committee for Animal Experiments of Juntendo University (Approval No. 2021211).

## Conflicts of Interest

The authors declare no conflicts of interest.

## Supporting information


**Figure S1:** In vitro DNA cleavage assay of the crRNA (a–e) targeting exon 7 of the mouse *Pah* gene. The 0.7‐kb PCR fragment of the mouse Pah gene was amplified by the primer set, mPah‐1 (5′‐CTAATGCTGCAACCTGGTAATACTGATCCTC‐3′) and mPah‐2 (5′‐TCAGCCAAGAGCTGGAATCATCCAGGTACATC‐3′) and purified following gel electrophoresis. The fragment (200 ng) was digested in a 20‐μL reaction containing 2 pmol Cas9 (Takara Bio Inc.), 2 pmol tracrRNA (Sigma‐Aldrich), and 2 pmol of the indicated crRNA (Sigma‐Aldrich) a to e at 37°C for 1 h. The reaction buffer consisted of 10 mM Tris–HCl (pH 7.5), 10 mM MgCl_2_, and 1 mM dithiothreitol. Electropherograms of the digested products, the undigested substrates (none), and the molecular DNA marker (M), are shown. The digested bands were quantified, and the digestion efficiencies were calculated as the ratios of the digested bands to the total DNA in the same lane. The digested efficiencies are shown at the bottom of each lane.

**Figure S2:** Analysis of Ax3DN and Ax6g AdVs containing the 3.7‐kb donor DNA and six gRNA expression units. (A) SmaI‐cleaved AdV genomes from the second stocks of three Ax3DN and Ax6g clones. (B, C) SmaI‐cleaved AdV genomes from the third, fourth, and fifth stocks of Ax3DN clone 2 (B) and Ax6g clone 2 (C). Bold blue arrows indicate the AdV DNA fragment containing the six multiplex gRNA units. Ladder bands of 6.1, 5.75, and 5.4 kb, corresponding to DNA fragments with at least four, three, and two gRNA units, respectively, were detected. These bands were visible just below the 6.8‐kb fragment and the topmost A band (evident in lanes labeled “4th” in Figures S2B and S2C). Lane m, DNA size markers; lanes 1 to 6, clone numbers; lane c, the parent cosmid containing the AdV genome. A, AdV fragment derived from the vector backbone.

**Figure S3:** Analysis of Ax3DN‐cut and Ax6g‐cut AdVs. Both AdVs contain the 3.7‐kb donor DNA, with six gRNA expression units located at the E4 site and two gRNA expression units at the E1 site. (A) SmaI‐cleaved AdV genomes from the second stocks of six Ax3DN‐cut clones. (B) SmaI‐cleaved AdV genomes from the third and fourth stocks of Ax3DN‐cut. (C) SmaI‐cleaved AdV genomes from the second stocks of six Ax6g‐cut clones. (D) SmaI‐cleaved AdV genomes from the third and fourth stocks of Ax6g‐cut. (E) SmaI‐cleaved AdV genomes from the fifth stocks of Ax3DN‐cut and Ax6g‐cut. (F) ClaI/NruI‐cleaved AdV genomes from the third, fourth, and fifth stocks of Ax6g‐cut. Bold blue arrows indicate the AdV DNA fragment containing the six (6g) and two (2g) gRNA units. Lane m, DNA size markers; lanes 1 to 6, clone numbers; lane c, the parent cosmid containing the AdV genome. A, AdV fragment derived from the vector backbone.

**Figure S4:** Comparison of knock‐in efficiency between the L‐region and R‐region for the knock‐in AdVs. Hepa1–6 cells were infected with the knock‐in AdVs (Ax6g, Ax3DN, Ax6g‐cut, and Ax3DN‐cut) and AxCBCas9/NC9 at MOIs of 100. Total cellular DNA was extracted 3 days postinfection and subjected to 30 cycles of PCR amplification using the primer sets shown in Figure 2 (LaF/4nR and 4nF/RaR). The PCR products were compared with those from a control DNA mixed with a known percentage of knocked‐in fragments.

**Figure S5:** In vitro knock‐in efficiency in Hepa1–6 cells analyzed by TIDER. Whole cell DNA samples (from the experiment shown in Figure 3B, total MOI 200) were amplified using the LaF/RaR primer set. The sequencing results of the 3.89‐kb PCR fragments were subjected to TIDER analysis. The knock‐in efficiency was consistent with the results obtained by the restriction enzyme digestion method shown in Figure 3B.

**Figure S6:** Enhancement of in vitro knock‐in efficiency by vanillin, L755507, and SCR7. HepG2 cells were infected with the Ax6g‐cut vector at an MOI of 100, together with AxCBCas9 at the same MOI. Vanillin, L755507, and SCR7 were added at the concentrations indicated. Total cellular DNA was extracted 3 days postinfection and subjected to PCR amplification using the primer set, LaF/sRaR, shown in Figure 5. The knock‐in efficiency was determined by digesting the 3.81‐kb PCR fragment with StuI (as shown in Figure 3). The 1.87‐kb and 1.94‐kb bands were quantified, and the efficiency was calculated as the ratio against the 3.81‐kb band in the same lane.

**Figure S7:** In vivo knock‐in efficiency in liver cells analyzed by TIDER. Whole cell DNA extracted from the livers of AdV‐treated mice (nos. 4–8) were amplified using the LaF/RaR primer set. The sequencing results of the 3.89‐kb PCR fragments were subjected to TIDER analysis. The knock‐in efficiency calculated by this method was below the detection limit.

## Data Availability

All data generated or analyzed during this study were included in this published article and its Supporting Information files.
